# Cryomilling of Isotope-Enriched Ti Powders for HIVIPP Deposition to Manufacture Targets for Nuclear Cross Section Measurement

**DOI:** 10.3390/ma16113926

**Published:** 2023-05-24

**Authors:** Sara Cisternino, Lucia De Dominicis, Liliana Mou, Juan Esposito, Claudio Gennari, Irene Calliari, Gaia Pupillo

**Affiliations:** 1 Legnaro National Laboratories, National Institute for Nuclear Physics (INFN-LNL), 35020 Legnaro, Italy; lucia.de.dominicis@lnl.infn.it (L.D.D.); liliana.mou@lnl.infn.it (L.M.); juan.esposito@lnl.infn.it (J.E.); gaia.pupillo@lnl.infn.it (G.P.); 2 Department of Industrial Engineering, University of Padova, 35131 Padova, Italy; claudio.gennari@unipd.it (C.G.); irene.calliari@unipd.it (I.C.); 3 Department of Physics and Astronomy, University of Padova, 35131 Padova, Italy

**Keywords:** isotopically enriched titanium, cryomilling, high energy vibrational powder plating, target manufacturing, material characterization, radionuclides

## Abstract

The realization of isotopically enriched Ti targets for nuclear cross-section measurements requires particular attention, from the starting material preparation up to the deposition technique. In this work, a cryomilling process was developed and optimized, aimed at reducing the size of ^49,50^Ti metal sponge as provided by the supplier (size up to 3 mm), to the optimal size of 10 µm, to fit the High Energy Vibrational Powder Plating technique used for target manufacturing. The optimization of the cryomilling protocol and the HIVIPP deposition using ^nat^Ti material was thus performed. The scarce amount of the enriched material to be treated (about 150 mg), the need to obtain a non-contaminated final powder and a uniform target thickness of about 500 µg/cm^2^ were taken into account. The ^49,50^Ti materials were then processed and 20 targets of each isotope were manufactured. Both powders and the final Ti targets produced were characterized by SEM-EDS analysis. The amount of Ti deposited was measured by weighing, indicating reproducible and homogeneous targets, with an areal density of 468 ± 110 µg/cm^2^ for ^49^Ti (*n* = 20) and 638 ± 200 µg/cm^2^ for ^50^Ti (*n* = 20). The uniformity of the deposited layer was also confirmed by the metallurgical interface analysis. The final targets were used for the cross section measurements of the ^49^Ti(*p*,x)^47^Sc and ^50^Ti(*p*,x)^47^Sc nuclear reaction routes aimed at the production of the theranostic radionuclide ^47^Sc.

## 1. Introduction

In the production of radionuclides for nuclear medicine applications, or for nuclear physics experiments, the use of an appropriate high-purity target is a crucial step. The selection of stable isotopes in suitable chemical forms, with the desired isotopic and chemical purities, is among the important considerations for radionuclide production. Generally, stable enriched isotopes are used to optimize the production yield of the radionuclide of interest while minimizing the co-production of contaminants [1,2]. However, due to the typically low natural isotopic abundance of these materials, they are much more expensive and provided by a few suppliers worldwide. Sometimes they are provided in powder form, without a uniform shape and size distribution. This may not be an issue when standard target manufacturing techniques are used, such as evaporation, rolling or electrodeposition because before these processes, the powder is melted in a bead, pressed in a pellet or it is dissolved in the electrolyte solution, respectively [1,2,3,4,5,6]. However, in some cases, such as isotopically enriched refractory metals (e.g., Ti), non-standard techniques for target manufacturing, requiring a fine and homogeneous powder particle distribution, have to be considered instead [1,7,8].

In recent years, R&D efforts towards the manufacture of high-purity targets to sustain radioisotope production for research purposes or for nuclear physics experiments are among the goals addressed by the LARAMED (LAboratory of RADionuclide for MEDicine) program currently running at the INFN-LNL [8,9,10,11,12,13]. These research activities are also part of the “Target Service” and “Radioisotopes for Medicine and Applied Physics Service” at the INFN-LNL Research Division [14] and of the EURO-LABS project [15], highlighting the importance of non-standard target preparation techniques and target characterization.

In particular, the REMIX project at INFN-LNL [16] studied an emerging and promising therapeutic radionuclide ^47^Sc, whose optimal nuclear production route is not yet defined [17,18,19,20]. Our group is investigating the proton-induced nuclear reactions on ^49^Ti and ^50^Ti targets up to 70 MeV, in collaboration with the ARRONAX facility [21], to determine the best irradiation parameters for ^47^Sc production to find the optimal radionuclide purity and to estimate the expected production yield [22,23,24]. Therefore, to conduct these studies (i.e., nuclear cross section measurements), ^49^Ti and ^50^Ti targets were necessary.

The High Energy Vibrational Powder Plating (HIVIPP) process was demonstrated as an interesting technique, especially for the deposition of refractory metals from powder form, and it is particularly advantageous because it guarantees negligible material waste during the process [7,25,26,27,28]. As reported by Skliarova et al. in [29], this technique has successfully led to the deposition of uniform layers of ^48^Ti onto Al substrates, allowing the use of these targets for nuclear cross-section measurements.

Therefore, in this work, the same HIVIPP technique was used to manufacture isotope-enriched ^49^Ti and ^50^Ti targets. However, according to Scanning Electron Microscopy (SEM) analysis, the ^48^Ti starting powder used in the previous work [29], had a uniform particle size dispersion in the range of 0.1–5.0 µm (as provided by the supplier). Instead, the only supplier in the world provides the isotope-enriched ^49,50^Ti materials in metallic powder form with a particle size in the range of 100 µm to 3 mm. From the first tests, this wide particle size range led to not uniform HIVIPP depositions. Therefore, it was clear that changes in the starting powder size would have a significant impact on the uniformity of the thickness distribution and the subsequent results of the nuclear cross-section measurements. Indeed, the HIVIPP deposit microstructure reflects the powder size; therefore, in order to have a uniform Ti layer with a thickness < 10 µm (i.e., ideal value for nuclear cross-section measurements) the powder size should be of the same order of magnitude or lower. Furthermore, from our experience with the HIVIPP technique, the use of particles with irregular shapes is preferable to using spherical particles. This is probably due to the fact that the not spherical particles (sharply curved) may more easily acquire higher kinetic energy during the HIVIPP process (under a static electric field) and thus more easily move in the quartz cylinder, having a higher chance to be implanted in the substrates.

Taking into account the non-homogeneous powder size of ^49,50^Ti (metallic sponge shaped), an additional process was mandatorily implemented in this work to obtain finer powder such as the one previously used for ^48^Ti material, which provided uniform areal density depositions with the HIVIPP process [29].

The ball-milling process, consisting of powder size reduction by the repeated impact of the powder particles between moving balls and container walls, is the most widely used technology in powder metallurgy for different applications. Several studies reported that the properties of powders, such as particle size and morphology, contamination and microstructure, would affect the processing as well as properties of the final products. The correlation between milling parameters and powder properties has been extensively studied for different materials in order to control and optimize the powder microstructure [30,31,32]. 

The repeated plastic deformation during milling leads to cold-welding, fracturing operations among the powder particles, and the generation of crystal defects, which in turn affect the structural changes in the powder. The temperature experienced by the powder during milling can also determine the nature of the final powder product. It was demonstrated that at low temperatures the dynamic recrystallization and grain growth are suppressed, thus the enhancement of grain refinement leads to an ultra-fine-grained microstructure within each powder particle [33,34,35,36,37,38,39,40]. An empirical explanation of the development of nanostructures is reported by Witkin [35]: during continued milling the increasing of dislocation density is followed by annihilation and recombination of dislocation with the formation of nanometer-scale sub-grains. In the end, the structure of the sub-grain is transformed into randomly oriented high-angle grain boundaries. 

In general, compared to conventional ball-milling at room temperature, ball milling at cryogenic temperatures (cryomilling) leads to an increased brittleness of the powder particles at such low temperatures (i.e., −196 °C in the case of Liquid Nitrogen), thus overcoming the trend of powder particles adhering to container walls, agglomerating and sintering to form large millimeter-sized particles. This leads to the production of finer particles and the milling time is reduced probably because of the suppression of the recovery effect at cryogenic temperatures [41]. Milling in direct contact with Liquid Nitrogen (LN) is known to cause nitrogen contamination in the material with a high affinity to N (i.e., Ti). Even if the diffusivity is reduced at low temperatures, severe deformation could trap nitrogen atoms in micropores [41,42]. As reported by Kozlík et al. [38] the intake of N in the Ti matrix makes the material more brittle, which may affect the mechanical behavior of the consolidated materials. In addition, other inevitable contaminations arise from the balls and milling tanks, which depend mainly on the milling intensity in terms of duration, material balls and jar size with respect to the starting material amount [36]. These contaminations could affect the purity and thus the performance of the final product.

In this work, the achievement of optimal manufacturing procedure (powder milling and subsequent deposition), aimed at obtaining the final target as free as possible from heavy metals impurities (<5 at.%), is mandatory for the success of correct nuclear cross section measurements. Other lighter elements, i.e., Si, O, N, etc., do not jeopardize the nuclear cross-section measurements. A cryomilling machine that guarantees the insulation between nitrogen and the material to be treated was used. The jar containing the material is hermetically closed in a dedicated jar and the LN is made to circulate externally in a dedicated jacket. The smallest stainless-steel jar and balls available were chosen, considering that the amount of ^49,50^Ti enriched materials available was considerably low, about 150 mg for each material. A detailed study with ^nat^Ti materials was performed in order to choose the optimal cryomilling protocol to obtain finer powder size while limiting contamination as much as possible. Particular attention was paid to the evaluation of the material losses after the process, in order to apply this protocol to the expensive isotopically enriched Ti materials. The aim is to have negligible losses to preserve precious materials. The powders obtained after each experiment were analyzed with SEM-EDS and used for the HIVIPP depositions, whose parameters were chosen to achieve the desired areal deposited density suitable for nuclear cross section measurements.

The aim of this work is to demonstrate that the cryomilling process can be used with metal-enriched materials without affecting their purity and that subsequently, the cryomilled powders can be used for the deposition of uniform layers onto metal substrates through the HIVIPP technique.

The experiments and first results are described hereafter, to underline that the entire technological development implemented in this work (the study of the cryomilling and HIVIPP parameter’s optimization) was essential to obtain suitable targets used for the specific application, i.e., nuclear cross section measurements for medical radionuclide production.

## 2. Materials and Methods

### 2.1. Materials

Metallic powder of ^49^Ti and ^50^Ti, respectively, 300 mg and 150 mg were purchased from the only provider in the world, i.e., the Oak Ridge National Laboratories (Tennessee, OR, USA). The datasheets of the isotope-enriched materials are shown in Tables S1 and S2. The original ^49,50^Ti materials had a metallic sponge shaped up to 3 mm, composed of powder particles welded together, probably due to the isotopic enrichment process. 

^nat^Ti sponge, 3–19 mm, 99.95% (metal basis) purchased from Alfa Aesar, was used as a starting material for the optimization of the powder preparation process (cryomilling) and the HIVIPP depositions to simulate the behavior of the isotopically enriched ^49,50^Ti. The optimized processes were then transferred to the enriched materials. 

Aluminum foils (25 μm thick, 99% purity) from Goodfellow were used as substrates (Ø 24 mm). Aluminum was a suitable material for the HIVIPP deposition and for the nuclear cross-section measurements [29]. 

### 2.2. Cryomilling Process: Instrumentation for Powder Preparation

The CryoMill machine used in this work was purchased by Retsch GmbH (Haan, Germany). The grinding jar is continually cooled down by liquid nitrogen (LN) from the integrated cooling system and the temperature of −196 °C is kept constant since the LN circulates through the system and is replenished by an autofill system (Dewar APOLLO Cryotherm). No direct contact of the sample with LN is ensured. The CryoMill grinding jar performs radial oscillations in a horizontal position. Pulverization of the sample material is caused by the high energy impact of the grinding balls on the sample at the rounded ends of the grinding jar. During ball milling, contamination with particles derived from milling debris is virtually unavoidable. To limit such contribution, the CryoMill machine supplier recommends that the volume of the grinding jar should be filled in by approximately one-third with the sample and one-third with the number of balls ensuring a free jar volume necessary for the free, random, balls. In this work, due to the very low sample amount available of isotopically enriched materials, the smallest available Stainless-Steel jar of 5 mL and the balls of Ø 7 mm were used.

About 150 mg of the cheapest ^nat^Ti under sponge form for each milling experiment, to mimic the behavior of ^49,50^Ti, were used. Different milling times and ball numbers were tested to find out the best recipe to obtain a powder size that fits the HIVIPP deposition process, leading to uniform targets.

After each milling experiment with ^nat^Ti, the jar was cleaned in an ultrasonic bath with dH_2_O for 60 min and dried with ethanol and compressed air. The balls were replaced with new ones. A Teflon o-ring (to ensure the closing of the jar) supplied by Retsch or a Kapton o-ring (about 700 µm thick) was used. 

The obtained Ti powder was recovered from the jar without scratching the walls, collected in a glass vial, weighted to quantify the losses, and analyzed by SEM to determine its size and by EDS for the identification of elemental traces.

New cleaned jars and balls were used for each enriched material to avoid cross-contamination and to guarantee the original enrichment level.

### 2.3. HIVIPP Deposition

Depositions were performed using the HIVIPP apparatus described by Cisternino et al. in [12]. Briefly, the set-up consists of a vacuum system able to reach 10^−7^ mbar without baking, a high voltage power supply SPELLMAN SL60N60/230 (Spellman High Voltage Electronic Corporation, Hauppauge, NY, USA) controlled with the LabView program properly designed to control the voltage and define the voltage ramp. The substrates (i.e., the Al foil in this work) are easily assembled on a dedicated sample holder made of stainless-steel electrodes and PEEK insulation parts. Two targets, named top (T) and bottom (B), are simultaneously obtained. The powder is inserted into a quartz cylinder of height 10 mm and internal Ø 14 mm, which is placed between the substrates.

**Figure 1:**
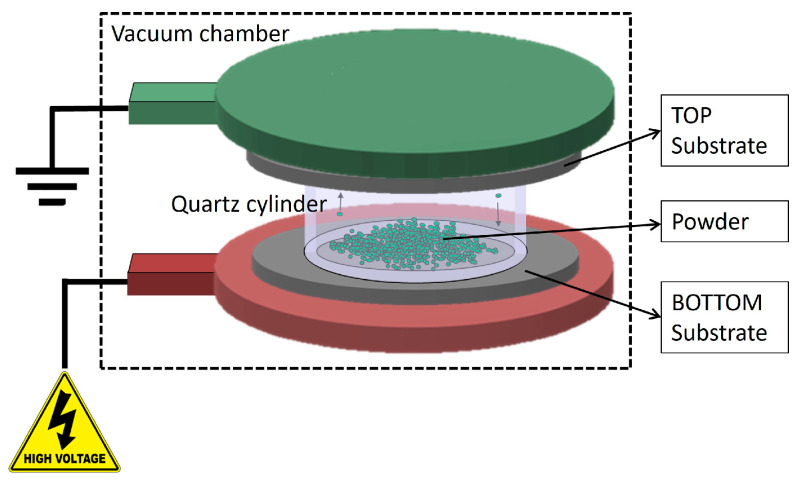
Schematic representation of the HIVIPP deposition technique.

When the vacuum level inside the chamber reaches approximately 1 × 10^−7^ mbar, negative voltage values of 10 kV, 12 kV and 15 kV were applied to power the bottom electrode through a 60 kV vacuum feedthrough. The top electrode was grounded and connected to the chamber. When the powder particles start to be charged, they move toward the electrodes of the opposite charge and the deposition occurs. A schematic representation of the HIVIPP process is shown in Figure 1.

The power supply keeps the current below 80 μA in order to avoid sparks and plasma generation inside the cylinder. The deposition process lasted from 10 h to 65 h.

To ensure the process reproducibility, the following steps were performed:

- weighing the Al substrates;
- loading about 20 mg of Ti powder in the quartz cylinder;
- placing the sample holder in the vacuum chamber and starting the vacuum system with the cylinder open;
- when the pressure reaches about 1 × 10^−7^ mbar, close the cylinder and start the deposition increasing the voltage following a set ramp until the desired maximum value;
- after deposition, carefully recovering the undeposited powder into a dedicated glass vial for subsequent reuse, and then cleaning the samples with compressed air to eliminate the negligible residuals of the not-attached powder.
- weighing the samples to calculate the amount deposited.

### 2.4. Characterization Analysis

The powders before and after each milling experiment and the target surface were analyzed using a Fei (ex Philips Amsterdam, The Netherlands) Scanning Electron Microscope SEM XL-30 and a Coxem CX-plus 200 electronic microscope (Yuseong-gu, Daejeon, Republic of Korea), coupled with the Brucker EDS probe (Billerica, MA, USA) at INFN-LNL.

The size particle range was estimated qualitatively by SEM image, using the slide gauge function, measuring the distance between two parallel tangents of the particle at an arbitrary angle. The bigger particles and the smaller particles are visually identified to determine the size range assuming that the analyzed material is representative of the entire material amount.

Powder weighing was performed by using a four digits analytical balance RADWAG (Radom, Poland), model AS220.R2. For the calculation of the areal mass thickness, the substrates were weighed prior and after the deposition by using a five digits balance Sartorius Semi-Micro Balance ME235S (Göttingen, Germany), readability 0.01 mg. On the basis of previous experiments, the uniformity of the deposits is guaranteed [12,29] thus, the Ti deposited thickness in µg/cm^2^ was calculated considering the mass deposited divided by the deposition area (calculated using the internal diameter of the quartz cylinder, 14 mm).

In order to study the deposit section as well as the interface between Ti and Al, a ^nat^Ti target prepared by using 15 kV for 66.5 h was cut and analyzed at the Department of Industrial Engineering (DII) at Padua University. The sample preparation consisted of mounting it in an epoxy resin (cold process, in vacuum to avoid bubbles) with fluorescent powder and then cutting it with a micro-cutter using a SiC blade. One half was polished using SiC paper of different grits (500–800-1200–4000) and polishing cloth with 6 μm and 1 μm colloidal suspension [43]. A thin gold layer was sputtered in order to make the surface conductive for SEM analysis using a Cambridge Leica Stereoscan LEO 440 Scanning Electron Microscope, equipped with a Philips PV9800 EDS probe.

## 3. Results and Discussions

### 3.1. Powder Preparation with Cryomilling Process

**Figure 2:**
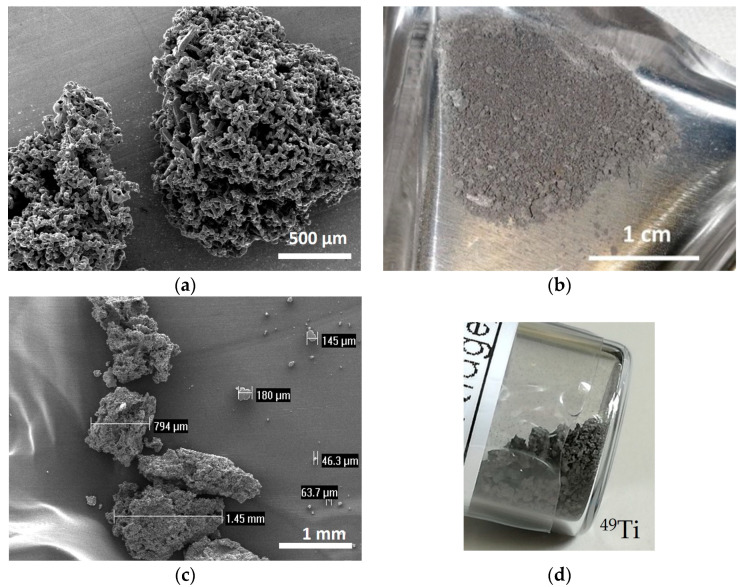
(**a**) SEM image of ^nat^Ti powder; (**b**) picture of ^50^Ti powder, (**c**) SEM image of ^49^Ti powder and (**d**) picture of ^49^Ti powder in the original glass vial.

A suitable method to mill the enriched Ti material, provided in a sponge-like shape, was developed. The following considerations were taken into account: (i) the amount of isotope-enriched powder was very low (about 150 mg) and its shape is sponge-like (100 μm–3 mm size range) as shown in Figure 2b–d; (ii) the possible contamination of heavy elements in the final powder should be within 5%, in order to be suitable for the final targets aimed at nuclear cross section measurements. The ^nat^Ti material, with a shape similar to that of the enriched one (Figure 2a), was used to mimic the isotopically enriched material for the preliminary tests to find the optimal cryomilling parameters. To better simulate the enriched materials, a similar amount of ^nat^Ti (i.e., 150 mg) was used for each milling experiment.

The detailed cryomilling (CM) parameters used with ^nat^Ti and the corresponding results are listed in Table 1. The CM machine allowed setting a custom milling cycle. In this work, it included 7 min of pre-cooling at 5 Hz, 3 min of CM at 30 Hz and intermediate cooling at 5 Hz (2 or 3 min). The powder size range obtained was deduced from SEM images. The powder lost corresponded to the powder remaining attached to the jar and balls.

**Figure 3:**
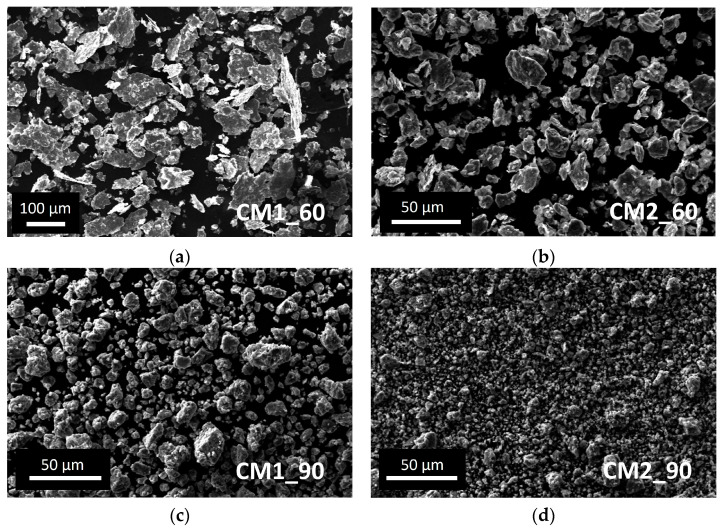
SEM images of ^nat^Ti powder obtained after the cryomilling experiments: (**a**) CM1_60; (**b**) CM2_60; (**c**) CM1_90; (**d**) CM2_90.

In Figure 3, the morphology of the powders with respect to the CM parameters is presented as a function of the milling time and the number of balls used. The flattened shape of the powder and the wide powder size range resulting from experiment CM1_60 is clearly visible. Adding another ball during the CM process, the powder shape resulted as irregular with a size between 5 and 30 µm (CM2_60). The effect of the milling time, using the same ball number is a considerable decrease in the powder size and a reduction in the powder size range (CM1_60 vs. CM1_90 and CM2_60 vs. CM2_90). The shape and the powder size range obtained in CM2_90 are considered to fit the HIVIPP requirements to achieve uniform deposition.

**Figure 4:**
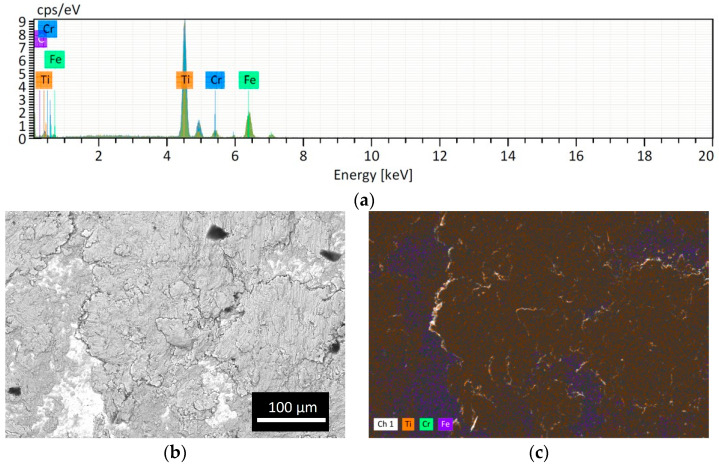
Analysis of the SS ball surface after CM1_90: (**a**) EDS spectrum; (**b**) BSE image and (**c**) EDS map. The presence of Ti attached is evident (orange spots).

**Figure 5:**
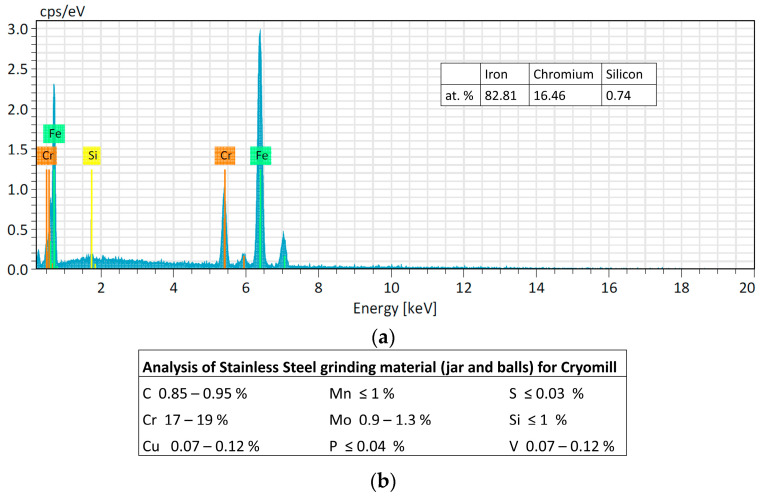
(**a**) Analysis of the intact SS ball. (**b**) Table of the analysis of Stainless Steel grinding material (jar and balls) for Cryomill machine provided by Retsch company.

However, as expected, the powder lost is higher if the obtained powder size is smaller because the smallest particles remained easily attached to the jar and balls wall as shown in Figure 4. The Cr peak comes from the ball material because it is made of Stainless Steel with Cr content >12%. The EDS analysis of the SS ball intact is in accordance with the datasheet of the grinding material provided by the Retsch company (Figure 5). 

**Figure 6:**
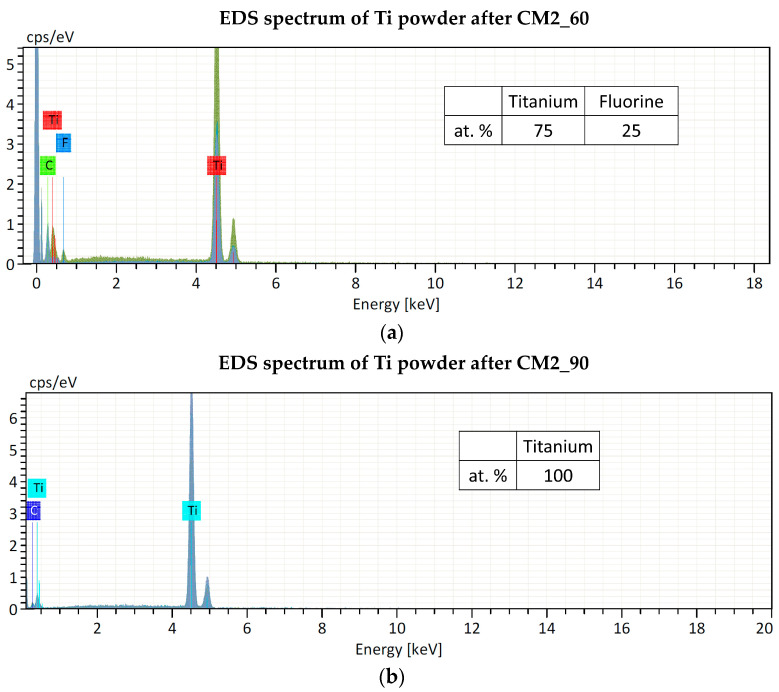
EDS spectra of Ti powder after cryomilling experiments: (**a**) CM2_60 and (**b**) CM2_90. The Carbon peak is due to the substrate where the powder was placed for the analysis.

Regarding contamination, the filling level of the jar is of crucial importance for the success of the grinding process. It is indeed essential to ensure that enough of the material to be ground is poured into the jar because during operation with a high frequency and with large diameter balls, an inadequate quantity of material for grinding will inevitably cause damage to the grinding jars and the balls. Only an adequate amount of material inside the grinding jar (about 1/3 of the jar volume) can serve as a protective layer between the balls and the surface of the grinding chamber, as explained in Section 2.2. As confirmation of this, preliminary experiments were carried out using the same amount of starting material (approx. 150 mg), but a larger stainless-steel jar (10 mL) and balls (12 mm) and a ZrO_2_ jar (25 mL) and balls (15 mm). The powder obtained was contaminated with Fe and ZrO_2_, respectively, thus it was not suitable for the nuclear cross-section measurements. The smallest stainless-steel jar available on the market has a volume of 5 mL. In this work, even if the material to be milled is too low to fill one-third of the jar volume (as recommended by the supplier to avoid contamination from the jar and balls material, see Section 2.2), no contamination from the jar and ball materials was found by EDS analysis. Only fluorine contamination, probably due to the Teflon o-ring used to seal the jar, was detected in the powder after the CM1_60 and CM2_60 experiments. In experiments CM2_90 and CM1_90, the o-ring was substituted with Kapton material, and no contaminations were found, as confirmed by the EDS spectra shown in Figure 6.

**Figure 7:**
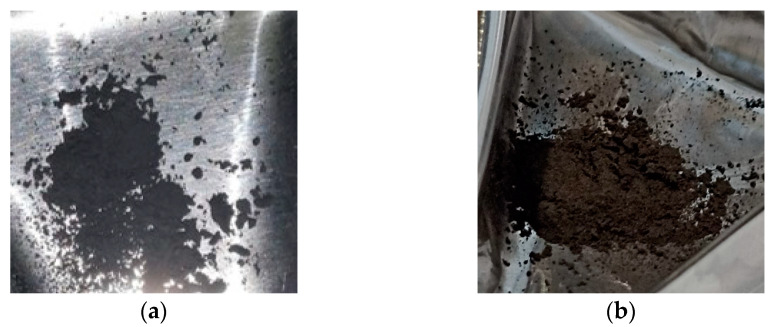
Pictures of the enriched ^49^Ti (**a**) and ^50^Ti (**b**) powders after the cryomilling process.

The CM parameters used in experiment CM2_90 led to the smallest powder size, fitting the HIVIPP deposition. Indeed, the shape and size of the powder are similar to those of ^48^Ti, which has already been successfully used for the realization of targets aimed at nuclear cross section measurements [29]. Therefore, ^49,50^Ti enriched materials were processed using the same parameters and the results are shown in Figure 7. Due to very few amounts of the expensive enriched materials, the ^49,50^Ti cryomilled powders were not analyzed by SEM-EDS to avoid an additional powder loss. However, the analyses of their HIVIPP depositions were performed, and the powder size (about <10 µm) and purity can be deduced. More details are described in Section 3.3.

As already observed with the ^nat^Ti material, the amount of powder recovered after cryomilling is lower with respect to the starting amount. In fact, from 160.3 mg and 147.9 mg of ^49^Ti and ^50^Ti, respectively, the available amounts for HIVIPP depositions were 125.8 mg (^49^Ti) and 111.9 mg (^50^Ti). The estimated loss was approximately 21.6% (^49^Ti) and 24.3% (^50^Ti) after the cryomilling treatment. However, it can be considered almost acceptable as the amount of powder available was sufficient for a consistent number of HIVIPP deposition experiments, considering the high efficiency of this technique.

### 3.2. ^nat^Ti Powder HIVIPP Depositions and Characterization

The HIVIPP experiments were performed with ^nat^Ti powder obtained after experiment CM2_90 and one deposition with CM1_90 powder. Instead, the ^nat^Ti powder obtained after CM1_60 and CM2_60 was not suitable for HIVIPP deposition. The parameters and the corresponding results, in terms of the Ti mass density deposited in µg/cm^2^, are reported in Table 2.

**Figure 8:**
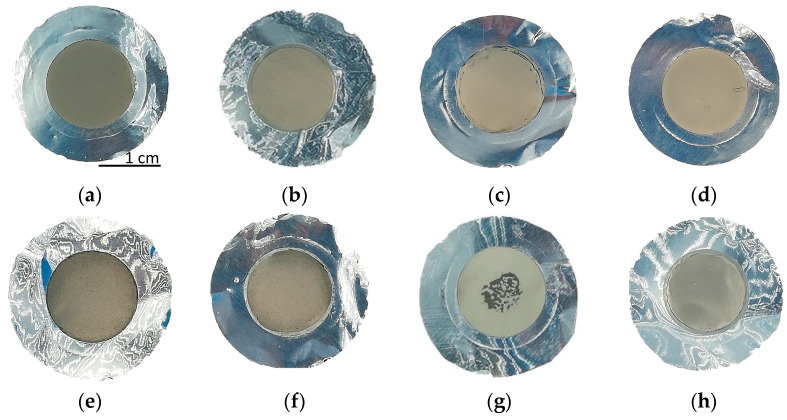
Picture of the ^nat^Ti targets: (**a**,**b**) #97 Top and Bottom; (**c**,**d**) #104 Top and Bottom; (**e**,**f**) #102 Top and Bottom; (**g**,**h**) #101 Top and Bottom. The deposition diameter is 14 mm.

Depositions with 10 kV for 10 h using the smallest powder resulted uniform and the thickness values were similar for the top and bottom samples (except for the exp. #97, probably because the bottom sample was cleaned with a brush). No process issues (e.g., discharge) occurred during the high-voltage application. On the contrary, by increasing the voltage up to 15 kV, some discharges which could cause non-uniformity, were observed (i.e., exp. #100 and #101). In Figure 8, the pictures of some representative ^nat^Ti targets are shown (#97, #104, #102 and #101).

**Figure 9:**
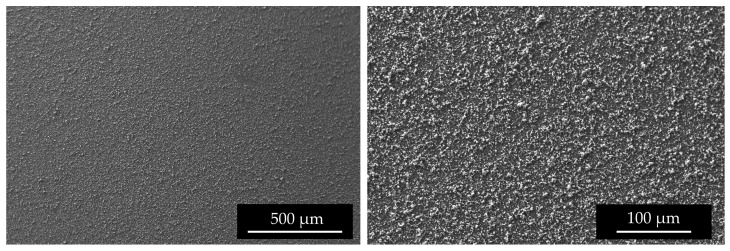
SEM surface images of #96 Top deposition at different magnitudes.

**Figure 10:**
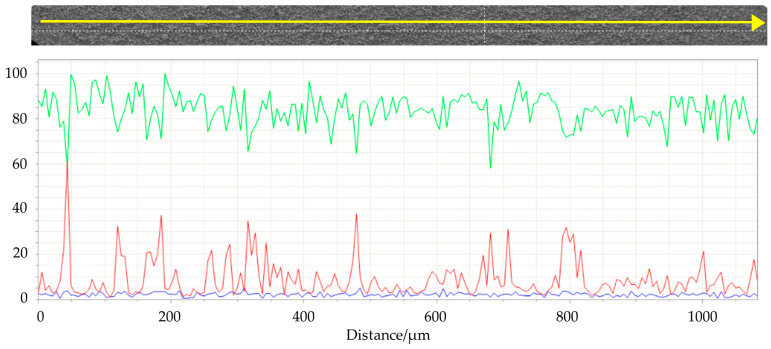
Linear EDS analysis of the surface of #99 Top target. The acquisition was carried out using 200 points with a distance of 5 µm. Green line: titanium; red line: aluminum; blu line: oxygen.

SEM images (Figure 9) of the top surface demonstrate the homogeneity of the deposit in the mm scale. In Figure 10 the linear EDS analysis of ^nat^Ti deposition is shown. The acquisition was carried out using 200 points with a distance of 5 µm. Beyond Ti, Al is detected by the instrument, especially at the point where the Ti layer is thinner.

The microstructure of the deposits reflects the powder size, as already observed in previous works [12,29].

**Figure 11:**
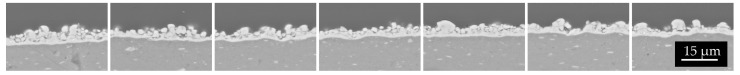
Several SEM images acquired at the same magnitude as the interface ^nat^Ti-Al.

**Figure 12:**
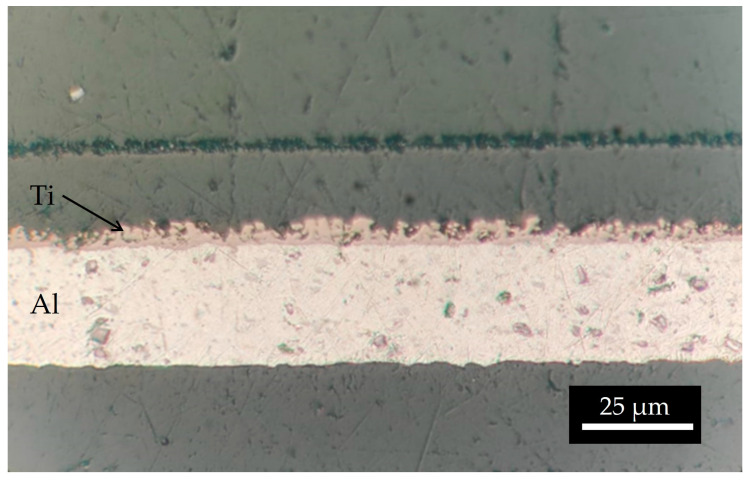
Optical microscope image of the interface ^nat^Ti-Al.

The mass density of the ^nat^Ti target realized using 15 kV and 66.5 h was 1906.5 µg/cm^2^, higher than the deposition performed for 10 h. The surface microstructure is similar to that of the samples with a lower thickness. As observed in the SEM interface images (Figure 11), the first layer of Ti powder appears well attached to the Al foil, then the larger particles create another layer, and some voids are visible. It can be deduced that the Ti film density is lower with respect to the bulk density. The roughness, deduced by the optical microscope image (Figure 12) and SEM images (Figure 11), is of the order of the powder size used (i.e., 5–10 µm) and some micro-inhomogeneities can be observed. However, on the mm scale, the targets are uniform, thus the targets can be used for nuclear cross section measurements because the proton beam hitting the Ti deposit has a diameter of 10 mm and the micro-inhomogeneity is negligible [29,44]. 

### 3.3. Enriched ^49^Ti and ^50^Ti HIVIPP Depositions and Characterization

Following preliminary experiments with ^nat^Ti, HIVIPP depositions with the enriched ^49^Ti and ^50^Ti were carried out. The HIVIPP parameters of each deposition and the corresponding thicknesses (Top and Bottom) in terms of the Ti mass density deposited, expressed in µg/cm^2^ are listed in Table 3. 

**Figure 13:**
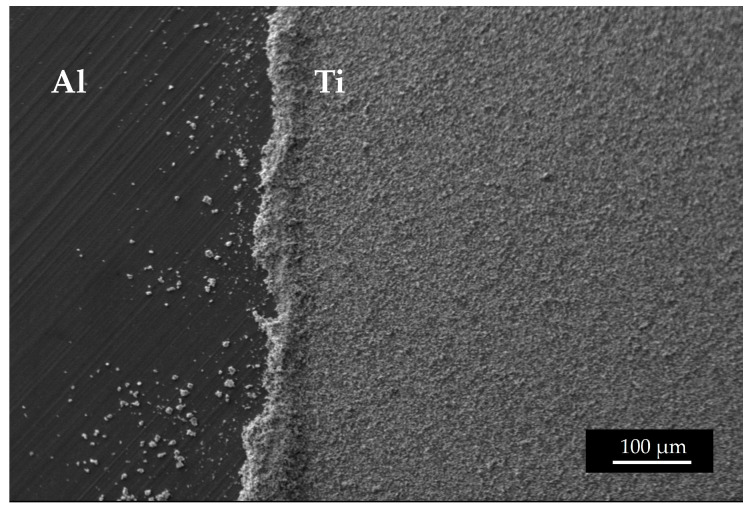
SEM image of the edge of a ^49^Ti target.

It must be noted that the weight precision could be affected by the uncertainties in determining the deposited area and by the edge powder accumulation or edge cleaning. In Figure 13a detail of the edge of a ^49^Ti target is shown. Considering the purpose of the targets realized in this work, the edge inhomogeneity is not relevant as they are cut with a punch to obtain a disc of 12 mm diameter to fit the target station size, where the samples are irradiated [29,44]. Since the exact quantification of the Ti deposited is a crucial ingredient for the nuclear cross section calculation, these targets were further accurately analyzed using the Elastic Backscattering Spectroscopy [45,46] following the same approach used in [29] and dedicated work is under preparation.

In summary, no. 20 ^49^Ti targets (corresponding to no. 10 experiments) were manufactured. The mass density values were similar for top and bottom targets. The exact values were 465.0 ± 108.9 µg/cm^2^ (*n* = 10) and 470.9 ± 117.7 µg/cm^2^ (*n* = 10) for the top and bottom targets, respectively. 

Ten experiments with ^50^Ti were performed corresponding to no. 20 targets as well. In this case, a slightly different mass density can be observed between top and bottom targets, 711.7 ± 284.3 µg/cm^2^ (*n* = 10) and 563.7 ± 103.5 µg/cm^2^ (*n* = 10), respectively.

**Figure 14:**
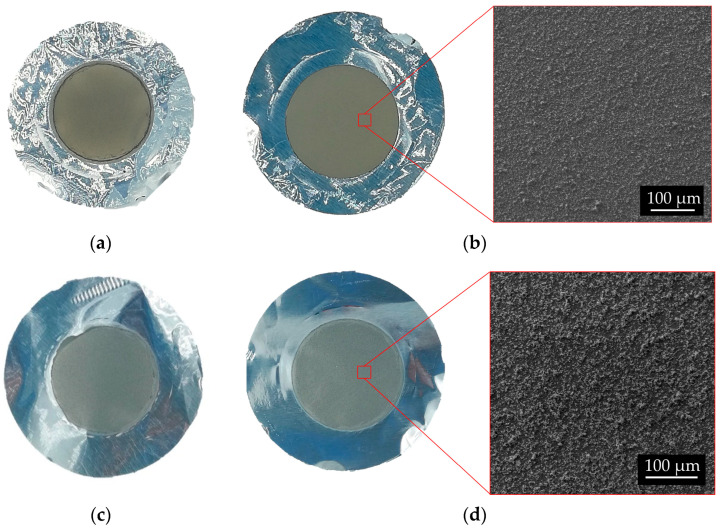
Picture of ^49^Ti and ^50^Ti targets and surface SEM images. ^49^Ti: (**a**) # 111 Bottom; (**b**) #111 Top. ^50^Ti: (**c**) #121 Bottom; (**d**) #121 Top.

In both cases, ^49^Ti and ^50^Ti, the thickness of the targets realized with powder recovered from a previous HIVIPP deposit was slightly higher (exp. #112–115 and exp. #124–127). This is probably due to the size of the recovered powder. Indeed, since smaller particles are deposited first, as described in [7,29], the particles not deposited (recovered and reused) are bigger, and therefore, the deposition thickness was higher. However, the uniformity of the deposits is confirmed for almost all ^49^Ti and ^50^Ti targets as shown in the pictures and SEM images in Figure 14. The targets can be compared with the ^48^Ti ones described in previous work [29].

**Figure 15:**
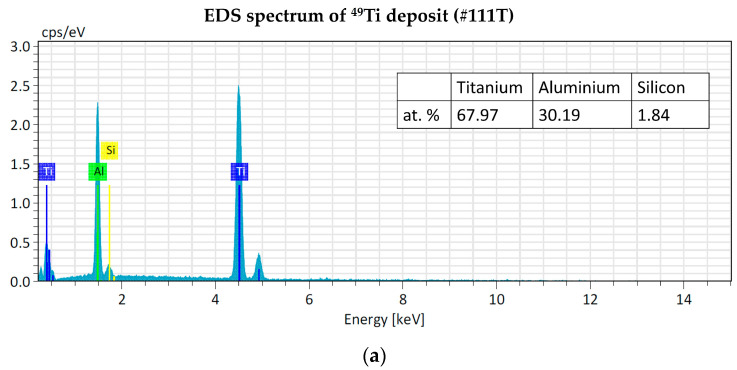

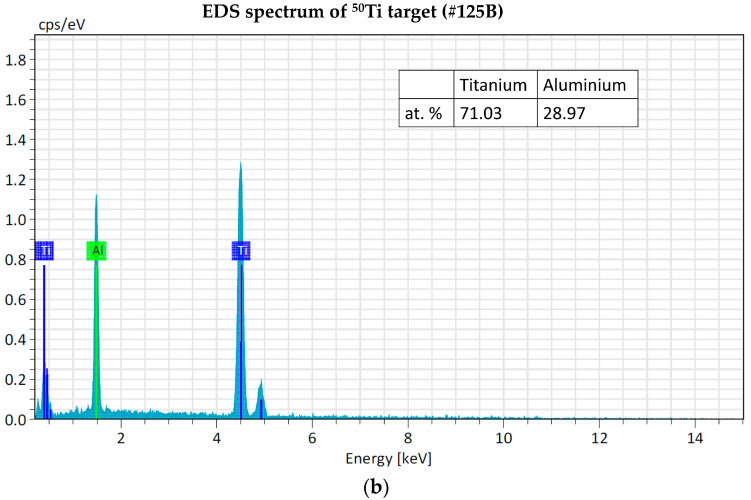
EDS analyses of ^49^Ti (**a**) and ^50^Ti (**b**) HIVIPP deposition.

The EDS analyses of ^49^Ti and ^50^Ti deposits confirm the purity of Ti powder after the cryomilling and HIVIPP depositions (Figure 15). The Si peak detected in the case of ^49^Ti is not due to the process performed in this work.

### 3.4. Efficiency of Cryomilling and HIVIPP Processes

The percentages of the efficiency for each deposition were similar to those obtained in the previous experimental campaign reported in [12], as shown in the summary Table 4.

**Figure 16:**
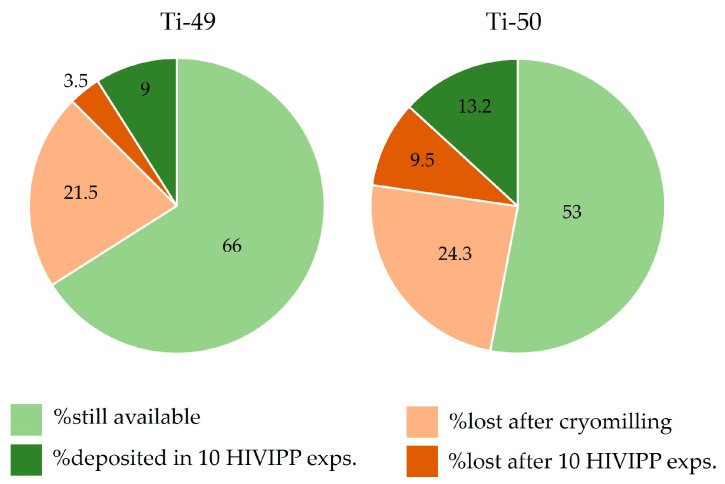
Percentage of the powder amount available, deposited, lost after cryomilling and lost after HIVIPP, respect to the total sponged-like material available.

In Figure 16, the percentage of the still available powders, the deposited powder and the losses with respect to the starting material amounts are reported. It can be observed that the most significant losses are due to the cryomilling process. However, these values appear to be important because the starting material amount was considerably low (i.e., about 150 mg). The amount of fine powders obtained after the cryomilling (more than 75% with respect to the total amount available for each isotope) was sufficient to prepare twenty targets. Therefore, the cryomilling losses can be considered a good compromise. Thanks to the possibility to re-use the not deposited powder after each HIVIPP deposition, more than 50% of each isotopically enriched material is still available and will be used for further experiments. 

## 4. Conclusions

In this work, the successful use of the cryomilling process was demonstrated for isotopically enriched Ti material with the aim of reducing the particle powder size to obtain uniform HIVIPP depositions as suitable targets for nuclear cross-section measurements. ^49^Ti targets produced in this work were used to measure, for the first time, the ^49^Ti (*p*,x) ^47^Sc nuclear cross section at high energy, up to 70 MeV.

From the authors’ experience, the problem of supply of other isotopically enriched materials in large agglomerates (e.g., ^52^Cr, ^64^Ni, etc.) is widespread; the cryomilling process could be a valid and versatile solution to make fine powders from precious materials, without contamination and with limited losses, for its use in different fields (e.g., target manufacturing for medical radionuclide production or nuclear physics experiments). However, it should be noted that each material has its own chemical and mechanical properties, thus the preliminary parameter optimization with the cheaper corresponding natural one is recommended. 

## Figures and Tables

**Table 1:** Details of the experimental cryomilling parameters and related results with ^nat^Ti.

Milling Parameters	Balls No.	Starting Material Amount [mg]	Results		Exp Name
			Powder Size Range ≈	% Powder Lost	
# cycle = 20 CM time = 60 min	1	143.6	30–200 µm	5%	CM1_60
	2	139.4	5–30 µm	15%	CM2_60
# cycle = 30 CM time = 90 min	1	161.7	2–20 µm	37%	CM1_90
	2	143.3	<10 µm	28%	CM2_90

**Table 2:** HIVIPP parameters and results of the experiments performed with ^nat^Ti. The * represents a not uniform deposition, and ^(u)^ means that the powder recovered from previous HIVIPP experiments was used.

Material	HIVIPP Parameters	Powder Size	Results [µg/cm^2^]		Exp #
			Top (T)	Bottom (B)	
^nat^Ti	10 kV; 10 h	<10 µm CM2_90	441.6	476.6	96
			476.6	260.0	97
			346.6	303.3	104 ^(u)^
			539.2	462.2	105 ^(u)^
		2–20 µm CM1_90	801.6	520.0	102
	15 kV; 10 h	<10 µm CM2_90	547.0	455.0	98
			563.3	563.3	99
			866.6 *	411.6	101
	15 kV; 21.5 h		1451.5 *	671.6	100 ^(u)^

**Table 3:** HIVIPP parameters and results of each experiment with ^49^Ti and ^50^Ti materials. The * represents a not uniform deposition, and ^(u)^ means that the powder recovered from previous HIVIPP experiments was used.

Enriched Material	HIVIPP Parameters	Results by Weighing [µg/cm^2^]		Exp #
		Top	Bottom	
^49^Ti	10 kV; 10 h	270.0	166.8	106
		435.5	498.3	107
		407.3	449.0	109
		404.6	465.4	110
		383.5	449.5	111
		536.7	457.1	113 ^(u)^
		494.0	546.0	114 ^(u)^
		485.3	547.0	115 ^(u)^
	12 kV; 15 h	649.9 *	541.6	108
		582.8 *	588.7	112 ^(u)^
^50^Ti	12 kV; 22.5 h	606.6	664.6	118
	12 kV; 10 h	517.8	455.0	119
		558.9	506.4	120
		444.1	433.3	121
		465.8	425.5	122
		554.6	566.3	123
		1075.1	719.3	124 ^(u)^
		940.2	638.7	125 ^(u)^
		979.2	623.9	126 ^(u)^
		977.1	604.4	127 ^(u)^

**Table 4:** Mean and standard deviation of the amount of powder, % efficiency and thickness (weighing) of the HVIPP experiments performed with ^49^Ti (n_e_ = 10), ^50^Ti (n_e_ = 10) and both (n_e_ = 20). The % were calculated following the equation described in [12]. (rec = recovered, dep = deposited; Eff = efficiency; n_e_ = number of HIVIPP experiment; *n* = number of targets -top and bottom-).

	Powder [mg]				%					Thickness
	Starting	Rec	Dep	Lost	Rec	Dep	Lost	Eff rec	Eff dep	[µg/cm^2^]
^49^Ti n_e_ = 10	20.0 ± 1.5	17.9 ± 1.4	1.4 ± 0.3	0.6 ± 0.4	89.8 ± 1.9	7.0 ± 1.6	3.2 ± 2.0	96.6 ± 2.1	69.9 ± 17.7	468.0 ± 110.4 (*n* = 20)
^50^Ti n_e_ = 10	19.1 ± 0.9	15.7 ± 1.3	1.0 ± 0.3	1.4 ± 0.4	82.2 ± 3.5	10.3 ± 2.9	7.4 ± 2.4	91.7 ± 2.6	58.1 ± 10.6	637.8 ± 200.2 (*n* = 20)
^49^Ti and ^50^Ti n_e_ = 20	19.5 ± 0.6	16.8 ± 1.6	1.2 ± 0.3	1.0 ± 0.5	86.0 ± 5.3	8.7 ± 2.4	5.3 ± 3.0	94.2 ± 3.4	64.0 ± 8.4	552.9 ± 120.1 (*n* = 40)

## Data Availability

Not applicable.

## Supplementary Materials

The following supporting information can be downloaded at: https://www.mdpi.com/article/10.3390/ma16113926/s1, Table S1: Datasheet of ^49^Ti material by Oak ridge. The spectrographic results reported herein are semi-quantitative estimates valid to one significant figure. Elements listed above without values were not detected or would calculate less than 10 ppm. This analysis reflects enrichment and impurity levels prior to conversion/fabrication (if applicable). Symbols: M—major; T—trace; l-interference, <—lessthan; <=—lessthan/equal to; ≈—approximately; nd—notdetected; no analyses made in all other cases; Table S2: Datasheet of ^50^Ti material by Oak ridge. The spectrographic results reported herein are semi-quantitative estimates valid to one significant figure. Elements listed above without values were not detected or would calculate less than 10 ppm. This analysis reflects enrichment and impurity levels prior to conversion/fabrication (if applicable). Symbols: M—major; T—trace; l—interference; <—lessthan; <=—lessthan/equal to; ≈—approximately; nd—notdetected; no analyses made in all other cases.
