# Supplementary material for: Cryomilling of Isotope-Enriched Ti Powders for HIVIPP Deposition to Manufacture Targets for Nuclear Cross Section Measurement
Source: Materials (Basel). 2023 May 24;16(11):3926. doi: 10.3390/ma16113926 (PMC10253755; doi:10.3390/ma16113926)
Supplement: Supplementary file 1 [file materials-16-03926-s001.zip › materials-2396281-supplementary.pdf]

Supplementary material

Table S1. Datasheet of ^{49}Ti material by Oak ridge. The spectrographic results reported herein are semi-quantitative estimates valid to one significant figure. Elements listed above without values were not detected or would calculate less than 10 ppm. This analysis reflects enrichment and impurity levels prior to conversion/fabrication (if applicable). Symbols: M - major; T - trace; l-interference, < - lessthan; <= - lessthan/equal to; ≈ - approximately; nd - notdetected; no analyses made in all other cases.

Element: Titanium
Simbol: Ti
Isotope: 49
Batch: 216901

ISOTOPIC ANALYSIS		
**Isotope**	**Atomic percent**	**Precision plus/minus**
46	0.2200	0.00500
47	0.2200	0.00500
48	2.71000	0.01000
49	96.2500	0.01000
50	0.6000	0.00500

SPECTROGRAPHIC ANALYSIS							
**Element:**	**ppm**	**Element:**	**ppm**	**Element:**	**ppm**	**Element:**	**ppm**
**Ag:**	<50T	**I:**		**Rh:**		**Tc:**	
**Al:**	<100T	**In:**	<500	**Ru:**		**Te:**	
**As:**		**Ir:**		**S:**		**Th:**	
**Au:**	<500	**K:**	<10	**Sb:**	<500	**Ti:**	M
**B:**	<100	**Li:**	BUFFER	**Sc:**		**Tl:**	
**Ba:**	<100	**Mg:**	<100T	**Se:**		**U:**	
**Be:**	<10	**Mn:**	<100	**Si:**	100	**V:**	<100
**Bi:**	<200	**Mo:**	<100	**Sn:**	<100	**W:**	<500
**Br:**		**N:**		**Sr:**	<100	**Zn:**	<500
**C:**		**Na:**	400	**Ta:**	<500	**Zr:**	<200
**Ca:**	<100	**Nb:**	<500	**LANTHANIDES and ACTINIDES**			
**Cd:**	<150	**Ni:**	<100T	**Am:**		**La:**	<200
**Cl:**		**O:**		**Bk:**		**Lu:**	<50
**Co:**	<100	**Os:**		**Ce:**	<1000	**Md:**	
**Cr:**	<10	**P:**		**Cf:**		**Nd:**	<500
**Cs:**	<500	**Pa:**		**Cm:**		**Np:**	
**Cu:**	400	**Pb:**	<200	**Dy:**	<1000	**Pr:**	
**F:**		**Pd:**		**Er:**	<50	**Pu:**	<500
**Fe:**	<100	**Pm:**		**Es:**		**Sm:**	<500
**Ga:**	<200	**Po:**		**Eu:**	<50	**Tb:**	<500
**Ge:**	<200	**Pt:**	<500	**Fm:**		**Y:**	<50
**Hf:**	<500	**Ra:**		**Gd:**	<200	**Yb:**	<20
**Hg:**	<500	**Rb:**	<200	**Ho:**	<500	**Tm:**	<500
		**Re:**					

Table S2. Datasheet of ^{50}Ti material by Oak ridge. The spectrographic results reported herein are semi-quantitative estimates valid to one significant figure. Elements listed above without values were not detected or would calculate less than 10 ppm. This analysis reflects enrichment and impurity levels prior to conversion/fabrication (if applicable). Symbols: M - major; T - trace; I - interference; < - lessthan; <= - lessthan/equal to; ≈ - approximately; nd - notdetected; no analyses made in all other cases.

Element: Titanium
Simbol: Ti
Isotope: 50
Batch: 103290

ISOTOPIC ANALYSIS		
**Isotope**	**Atomic percent**	**Precision plus/minus**
46	1.6900	0.05000
47	1.2900	0.05000
48	12.5100	0.20000
49	1.4100	0.05000
50	83.1000	0.20000

SPECTROGRAPHIC ANALYSIS							
**Element:**	**ppm**	**Element:**	**ppm**	**Element:**	**ppm**	**Element:**	**ppm**
**Ag:**	<50	**I:**		**Rh:**		**Tc:**	
**Al:**	<200T	**In:**		**Ru:**		**Te:**	<500
**As:**		**Ir:**		**S:**		**Th:**	
**Au:**	<500	**K:**	<100	**Sb:**	<500	**Ti:**	M
**B:**	<100	**Li:**	<50	**Sc:**		**Tl:**	
**Ba:**	<100	**Mg:**	<100T	**Se:**		**U:**	
**Be:**	<10	**Mn:**	<100	**Si:**	<=100	**V:**	<200
**Bi:**	<200	**Mo:**	<100	**Sn:**	<200	**W:**	1000
**Br:**		**N:**		**Sr:**	<100	**Zn:**	<500
**C:**		**Na:**	1000	**Ta:**	<500	**Zr:**	<200
**Ca:**	100	**Nb:**	<500	**LANTHANIDES and ACTINIDES**			
**Cd:**	<500	**Ni:**	<200	**Am:**		**La:**	<200
**Cl:**		**O:**		**Bk:**		**Lu:**	<50
**Co:**	<200	**Os:**		**Ce:**	<1000	**Md:**	
**Cr:**	<200	**P:**		**Cf:**		**Nd:**	<500
**Cs:**	<500	**Pa:**		**Cm:**		**Np:**	
**Cu:**	200	**Pb:**	<200	**Dy:**	<1000	**Pr:**	
**F:**		**Pd:**		**Er:**	<50	**Pu:**	
**Fe:**	500	**Pm:**		**Es:**		**Sm:**	<500
**Ga:**	<200	**Po:**		**Eu:**	<50	**Tb:**	<500
**Ge:**	<200	**Pt:**	<500	**Fm:**		**Y:**	<50
**Hf:**		**Ra:**		**Gd:**	<200	**Yb:**	<20
**Hg:**	<500	**Rb:**	<200	**Ho:**	<500	**Tm:**	<500
		**Re:**					
